# Assessing the utility of SoilGrids250 for biogeographic inference of plant populations

**DOI:** 10.1002/ece3.10986

**Published:** 2024-03-11

**Authors:** Tony Miller, Christopher B. Blackwood, Andrea L. Case

**Affiliations:** ^1^ Department of Biological Sciences Kent State University Kent Ohio USA; ^2^ Department of Plant, Soil, and Microbial Sciences Michigan State University East Lansing Michigan USA; ^3^ Department of Plant Biology Michigan State University East Lansing Michigan USA

**Keywords:** digital soil model, edaphic niche properties, plant species distribution modeling, SoilGrids

## Abstract

Inclusion of edaphic conditions in biogeographical studies typically provides a better fit and deeper understanding of plant distributions. Increased reliance on soil data calls for easily accessible data layers providing continuous soil predictions worldwide. Although SoilGrids provides a potentially useful source of predicted soil data for biogeographic applications, its accuracy for estimating the soil characteristics experienced by individuals in small‐scale populations is unclear. We used a biogeographic sampling approach to obtain soil samples from 212 sites across the midwestern and eastern United States, sampling only at sites where there was a population of one of the 22 species in *Lobelia* sect. *Lobelia*. We analyzed six physical and chemical characteristics in our samples and compared them with predicted values from SoilGrids. Across all sites and species, soil texture variables (clay, silt, sand) were better predicted by SoilGrids (*R* ^2^: .25–.46) than were soil chemistry variables (carbon and nitrogen, *R* ^2^ ≤ .01; pH, *R* ^2^: .19). While SoilGrids predictions rarely matched actual field values for any variable, we were able to recover qualitative patterns relating species means and population‐level plant characteristics to soil texture and pH. Rank order of species mean values from SoilGrids and direct measures were much more consistent for soil texture (Spearman *r*_S_ = .74–.84; all *p* < .0001) and pH (*r*_S_ = .61, *p* = .002) than for carbon and nitrogen (*p* > .35). Within the species *L. siphilitica*, a significant association, known from field measurements, between soil texture and population sex ratios could be detected using SoilGrids data, but only with large numbers of sites. Our results suggest that modeled soil texture values can be used with caution in biogeographic applications, such as species distribution modeling, but that soil carbon and nitrogen contents are currently unreliable, at least in the region studied here.

## Graphical abstract

We used a biogeographic sampling approach to test the ability of SoilGrids data to recapitulate known relationships between soil variables and distributions of 22 plant species. SoilGrids predictions rarely matched actual field values, but we were able to recover qualitative patterns relating plant characteristics to soil texture and pH. Our results suggest that modeled soil texture values can be used with caution in biogeographic applications such as species distribution modeling, but that soil carbon and nitrogen contents are currently unreliable.

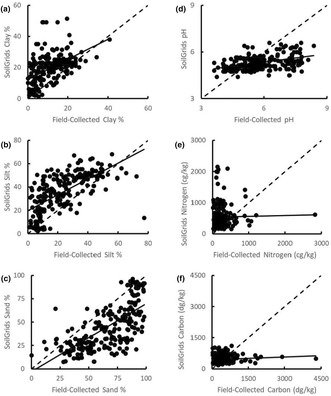

## 1 INTRODUCTION

Characterizing species distributions in geographic and environmental space can help us understand a species' niche, evolutionary history, and potential for interactions with co‐occurring species (Elith & Leathwick, 2009; Kozak et al., 2008; Pollock et al., 2014). One important component of predicting a species distribution is the inclusion of ecologically relevant predictors (Dormann, 2007; Mod et al., 2016). Modeled climate data has a long history of use in ecological modeling but for plant distributions, incorporating soil characteristics can further improve model accuracy (Dubuis et al., 2013; Figueiredo et al., 2018; Roe et al., 2022; Thuiller, 2013; Velazco et al., 2017; Zuquim et al., 2020). The inclusion of soil data has created the need to enhance the quality and availability of data on soil characteristics on a global scale.

To incorporate accurate soil data into ecological and biogeographic inference of plant species, soil characteristics would ideally be measured from cores collected at presence points across the full species range. Predictions for soil characteristics derived from digital soil maps may be useful substitutes, reducing the labor and cost of direct soil core analysis at range‐wide scales, as well as providing interpolated soil data for areas with limited accessibility (Grunwald et al., 2011; McBratney et al., 2003; Minasny & McBratney, 2016). The International Soil Reference Information Centre (www.isric.org) developed SoilGrids as a global collection of model‐predicted soil data for ease of use in a variety of settings, including soil erosion, food and water security, and modeling biodiversity and effects from climate change (Hengl et al., 2014; Poggio et al., 2021). The newest version of SoilGrids combined machine learning, 150,000 soil profiles for training, and 158 environmental covariates to provide global predictions at a scale of 250 × 250 m (Hengl et al., 2017). Comparing cross‐validation measures, *R* ^2^ values ranged from 56% (coarse soil fragments) to 83% (soil pH) across different soil variables (Hengl et al., 2017). However, the utility of SoilGrids data needs additional validation for its appropriateness in the development of species distribution models, particularly for low‐abundance plant species that are moderate habitat specialists.

The use of digital soil maps for biogeographic applications comes with clear limitations. First, the accuracy for modeling each soil characteristic varies, such that some soil variables will be more reliable than others (Poggio et al., 2021). Along with issues of model accuracies, there are scaling issues associated with the soil environment. For instance, climatological conditions are likely to be quite similar at the local scale (e.g., 1 km^2^ or smaller), whereas soil conditions can exhibit substantial heterogeneity at much finer scales (Heuvelink & Webster, 2001; Malone et al., 2017). Fine‐scale variation in soil characteristics created by microtopography and hydrology would not be captured in 250 × 250 m grid cells, and this is still much larger than the scale experienced by individual plants or even whole populations. Furthermore, SoilGrids does not predict soil conditions at locations with surface water or in cities (Poggio et al., 2021), potentially yielding missing or inaccurate data for wetland and aquatic habitats, even where plants of interest are dominant within the community.

To test the utility of SoilGrids specifically for biogeographic inference, we focused on a clade of wildflowers with highly variable geographic distributions and habitat types, including wetland and emergent aquatic species. *Lobelia* sect. *Lobelia* L. (Campanulaceae) is composed of 24 herbaceous species native to North and Central America. Some species are widespread across the eastern United States and Canada, while other species are found in only a few states (Biota of North America, BONAP, Kartesz, 2015; Spaulding & Barger, 2016). This clade provides an opportunity to document potential scaling effects, as species frequently co‐occur and appear to be separated into different microhabitat conditions within 250 m (unpub. data). One species, *Lobelia siphilitica* L., permits assessment of how soil conditions relate to trait variation among populations within a species. *Lobelia siphilitica* is composed of two sexes—females and hermaphrodites—which are readily observable in the field. Females vary dramatically in their frequency among *L. siphiltica* populations, and field data indicate that both population size and population sex ratio vary with soil conditions (Hovatter, 2008; Hovatter et al., 2013).

We tested how estimates from SoilGrids compared with soil data collected in the field at sites hosting *Lobelia* populations. The questions addressed here focus on: (1) the accuracy of SoilGrids estimates in habitats occupied by a set of closely related plants and (2) whether modeled soil values from SoilGrids lead to different inferences about species distributions and ecology compared to direct measurements on soils collected in situ. First, we determined how soil physical and chemical variables from SoilGrids compare to soil samples collected at sites hosting *Lobelia* populations. Second, we looked for associations between deviations of SoilGrids from measured field data and particular conditions (proximity to a water body or ecoregion). Third, we used two datasets to examine the extent to which SoilGrids data would be useful in understanding the biogeography of *Lobelia*. We collected and analyzed field soil from 22 *Lobelia* species at 212 population sites across the midwestern and eastern United States. We compared direct measures of soil characteristics to modeled SoilGrids data to test whether: (i) modeled SoilGrids data could predict patterns in average edaphic conditions among 22 *Lobelia* species, and (ii) in polymorphic *L. siphilitica*, whether data from SoilGrids could predict relationships between soil conditions and population sex ratios.

## 2 MATERIALS AND METHODS

### 2.1 Soil field data

In the summers of 2017 and 2021, we visited a total of 212 populations of 22 *Lobelia* species across the midwestern to eastern United States and Canada (Table S1), where we collected soil samples and GPS coordinates. Potential populations were identified from personal communications and using the Southeast Regional Network of Expertise and Collections (SERNEC, 2022). After removing any Oi horizon, soil samples were collected from the top 10 cm of soil underneath individual *Lobelia* plants (five samples per site, or from each plant if there were fewer than five present), which were bulked for analysis by population site and species. Distances between bulked soil samples ranged from 1 to 30 m. Population sizes ranged widely by site and species, from single plants to over 1000 individuals. Although most species prefer moist habitats, specific habitat conditions range widely among species and sites, including roadsides, upland forests, bogs, prairies, riparian areas, and near‐shore lacustrine habitats (Spaulding & Barger, 2016). Soil samples were allowed to air dry before passing through a 2 mm sieve, leaving only the fine‐earth fractions (sand, silt, and clay). pH was measured using a 1:2.5 mass ratio of soil to water. Percent carbon and nitrogen were measured using an elemental analyzer (Costech Analytical, Santa Clarita, USA). For texture analysis, sieved soils were first pretreated with 30% hydrogen peroxide to remove organic matter, and then analyzed using a laser‐diffraction particle size analyzer (Mastersizer 2000; Malvern Panalytical, UK). Soil aggregates were added to distilled water and broken up with 1 min of ultrasonication. We used a protocol measuring the texture distribution of three subsamples, each of which reached a laser obscuration value between 12% and 16%, and obtained the mean distribution of subsamples. As laser diffraction measurements underestimate clay and overestimate silt fractions in soil compared to the sedimentation method, we applied a correction factor as described in Di Stefano et al. (2010), which was confirmed for our laboratory (Figure S1), multiplying the clay fraction 1.9× and subtracting the resulting difference from the silt fraction.

### 2.2 SoilGrids data

Using population GPS coordinates, SoilGrids250 data were obtained for pH, carbon, nitrogen, and each of the three fine‐earth fractions (sand, silt, and clay). The data were accessed directly from the SoilGrids website in December 2022 (Poggio et al., 2021). In some cases, GPS coordinates landed in a grid cell with no SoilGrids data. In these cases, we used the nearest grid cell with data.

Because our in situ soil samples included the top 10 cm, we averaged SoilGrids layers for the surface horizon (0–5 cm) and the first subsurface horizon (5–15 cm) for our analyses using equal weights for each horizon. The 0–5 and 5–15 cm layers were strongly correlated for clay, sand, silt, and pH (*r* > .98), while the correlation between layers was weaker for nitrogen (*r*: .8) and weakest for carbon (*r*: .5). To further explore this, we conducted separate regressions comparing the field data with each individual horizon, and the results were similar as the average values (Table S2).

### 2.3 Comparison of SoilGrids predictions to field‐collected soil measurements

To investigate the relationship between SoilGrids data and field‐collected data, we conducted linear regressions for each variable using field‐collected measurements as the independent variable. We then examined goodness‐of‐fit measurements (*R* ^2^), slopes, root mean squared error (RMSE), and mean bias error (MBE) to determine agreement between SoilGrids predictions and observations obtained in the field. RMSE and MBE are expressed in the same units as the response variable (here, SoilGrids values). RMSE is used in comparing measured values with predicted values by using the square root of the sum of the squared residuals of the model. MBE, on the other hand, calculates the mean of the residuals and indicates whether variables are under‐ or over‐predicted.

### 2.4 Investigating environmental correlates of deviations between field and modeled data

The difference between measured and modeled values was calculated by subtracting SoilGrids values from field values. We then tested for associations between these SoilGrids‐measured differences and two environmental variables: proximity of the sample site to water bodies and ecoregion designation. Some GPS coordinates for populations near water bodies had no corresponding data from SoilGrids due to issues like shifting boundaries of water bodies. Sites close to water bodies may also be affected by flooding and hydrology that vary over small scales (i.e., a few meters). Thus, we tested whether the distance of a population to a water body affected the magnitude of SoilGrids‐measured differences. Water body data were obtained from the National Hydrography Dataset managed by the United States Geological Survey (USGS, 2019). We used QGIS 3.6 to determine the distance a population point was from the nearest body of water (QGIS.org, 2019). Linear regressions were used to investigate whether larger SoilGrids‐measured differences were associated with distance to the nearest water body.

We also used ecoregions to see if SoilGrids‐measured differences were associated with our sampling points being embedded in any particular habitat conditions. Data on ecoregions were obtained from the US Environmental Protection Agency (Omernik, 1987; Omernik & Griffith, 2014). We conducted the analyses using level‐2 ecoregions because many sampled populations fell into a single category of level‐1 ecoregions (eastern temperate forests; Table S2). To test for significant differences in SoilGrids‐measured differences across ecoregions, we used the non‐parametric Kruskal–Wallis one‐way ANOVA followed by the Steel–Dwass pairwise comparison method that controls for multiple comparisons and is robust to imbalanced sampling (Morley, 1982; Neuhäuser & Bretz, 2001).

### 2.5 Inferring ecological relationships between soil conditions and lobelia species

The utility of SoilGrids data for inferring soil conditions at *Lobelia* population sites was tested using two approaches. First, for each of 22 *Lobelia* species, we calculated the mean and standard error of field soil measurements and SoilGrids modeled data for each soil characteristic. Species were then ranked by mean field soil measurement to determine whether the ranking according to SoilGrids data would be consistent with measured habitat values. This procedure was used to see if SoilGrids could capture ecologically relevant but very broad, qualitative characteristics of the dataset without influence of outliers or noise introduced by individual site data. Congruence of species ranks was assessed by a Spearman rank correlation test (Spearman correlation shown below as *r*_S_).

Second, to compare how SoilGrids and field‐collected data associated with *L. siphilitica* population sex ratios, we conducted Spearman rank tests between each soil characteristic and the percent females in a population. This dataset was confined to 30 populations for which we had obtained both soil samples and population sex ratios for *L. siphilitica*. Sex ratios were calculated by sexing and counting all female and hermaphrodite plants at each site and are reported here as the percent of all censused plants that were female. In a second analysis, we used an expanded set of population sex ratios at 195 sites where *L. siphilitica* sex ratios and GPS coordinates had been recorded in situ, but no physical soil samples were available. This latter analysis was done to determine whether the associations between population female frequency and soil characteristics known from empirical measurements (*n* = 30) could be recovered by using modeled SoilGrids variables with an increased sample size. As sex‐ratio data are non‐normally distributed, we used Spearman rank tests. All statistics were calculated using JMP Version 14 (JMP Statistical Discovery; SAS Institute, Cary, NC, USA). Soil data was extracted using QGIS 3.6 (QGIS.org, 2019).

## 3 RESULTS

### 3.1 Accuracy of SoilGrids—Soil physical characteristics

**FIGURE 1:**
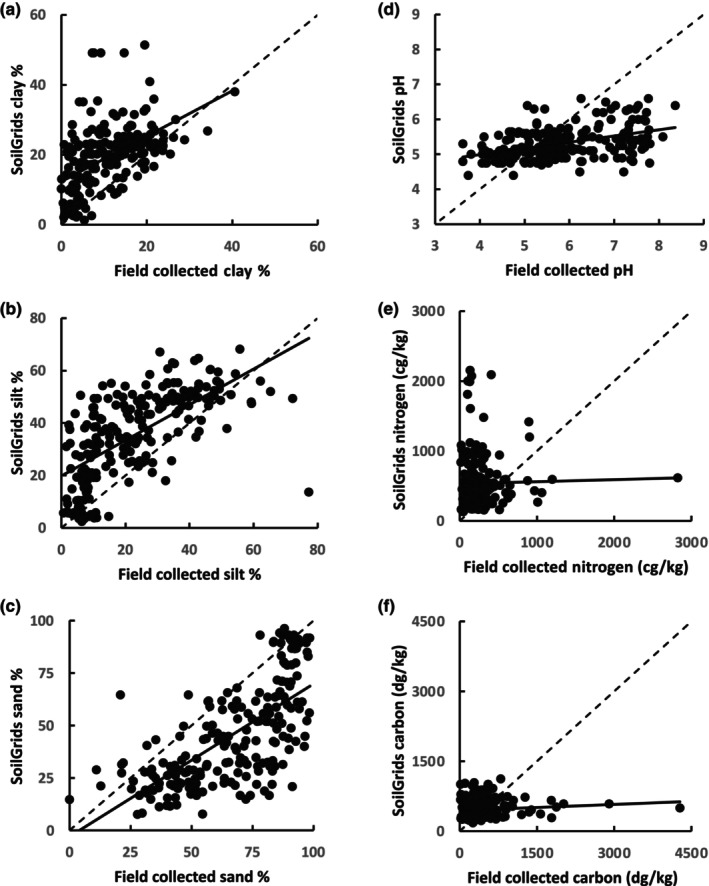
Relationships between field‐collected soil measurements and predicted soil measurements from SoilGrids. Solid lines represent relationships between the field‐collected data with the SoilGrids predicted data. Dashed lines represent a 1:1 line, which would be expected if the field collections and predictions have perfect agreement. (a) Clay (*R* ^2^: .25; Slope: 0.65 ± 0.08; *p* < .01; RMSE: 12; MBE: 8), (b) silt (*R* ^2^: .42; Slope: 0.67 ± 0.05; *p* < .01; RMSE: 18; MBE: 12), (c) sand (*R* ^2^: .46, Slope: 0.73 ± 0.05; *p* < .01; RMSE: 28; MBE: −21), (d) pH (*R* ^2^: .19; Slope: 0.17 ± 0.02; *p* < .01; RMSE: 1.1; MBE: −0.45), (e) nitrogen (*R* ^2^: .0004; Slope: 0.030 ± 0.1; *p* = .07; RMSE: 525; MBE: 280), (f) carbon (*R* ^2^: .01; Slope: 0.044 ± 0.03; *p* = .13; RMSE: 472; MBE: 19).

The estimated particle‐size fractions from SoilGrids were all positively correlated with the corresponding measurement from field‐collected soils (Figure 1). Of the three texture variables analyzed, the weakest relationship was in the clay fraction (Figure 1a, *R* ^2^: .25). Silt fractions and sand fractions showed relatively strong relationships between SoilGrids predictions and field‐collected data (Figure 1b,c, *R* ^2^: .42 & .46, respectively). Clay and silt fractions tended to be over‐estimated, as many of the data points fell above the 1:1 line (MBE: 8.5% and 12.3%, respectively; Figure 1). Sand fractions were under‐estimated, with most points falling below the 1:1 line (MBE: −21%). Overall, SoilGrids texture predictions were most accurate for soils with relatively high clay and silt but low sand (closest to the 1:1 line in Figure 1).

### 3.2 Accuracy of SoilGrids—Soil chemical characteristics

The soil pH from field‐collected soils had a weak, positive relationship with SoilGrids pH predictions (Figure 1d, *R* ^2^: .19). The range of SoilGrids pH values was much smaller (ranging from 4.4 to 6.6) than for field soils (ranging from <4 to >8). SoilGrids tended to over‐estimate pH for soils with pH below 5 and under‐estimate pH above 5.

For nitrogen and carbon, there was no relationship between field data and predicted data from SoilGrids (Figure 1e,f, *R* ^2^ < .01, and *R* ^2^: .01, respectively). The relationship was not improved by removing outliers (identified using the quantile range method in JMP), or examination of carbon to nitrogen ratio (*R* ^2^ < .01).

### 3.3 Investigating environmental correlates of variation between field and modeled data

The distance to the nearest water body did not account for discrepancies between field and SoilGrids data for any of the soil variables analyzed (*p* > .4 for each variable). Across ecoregions, we found significant differences for all variables of interest (Figure S2). Of note is that mean carbon SoilGrids‐measured differences can either be positive or negative depending on which ecoregion the soil core was collected. The SoilGrids‐measured differences for nitrogen were lowest in the southeast USA plains (Figure S2 panel e). However, even when conducting linear regression using only the southeast USA plains populations, the relationship for nitrogen concentration in the field and predicted from SoilGrids was still not significant (*R* ^2^ < .01).

### 3.4 Inferring ecological relationships between soil conditions and lobelia species

**FIGURE 2:**
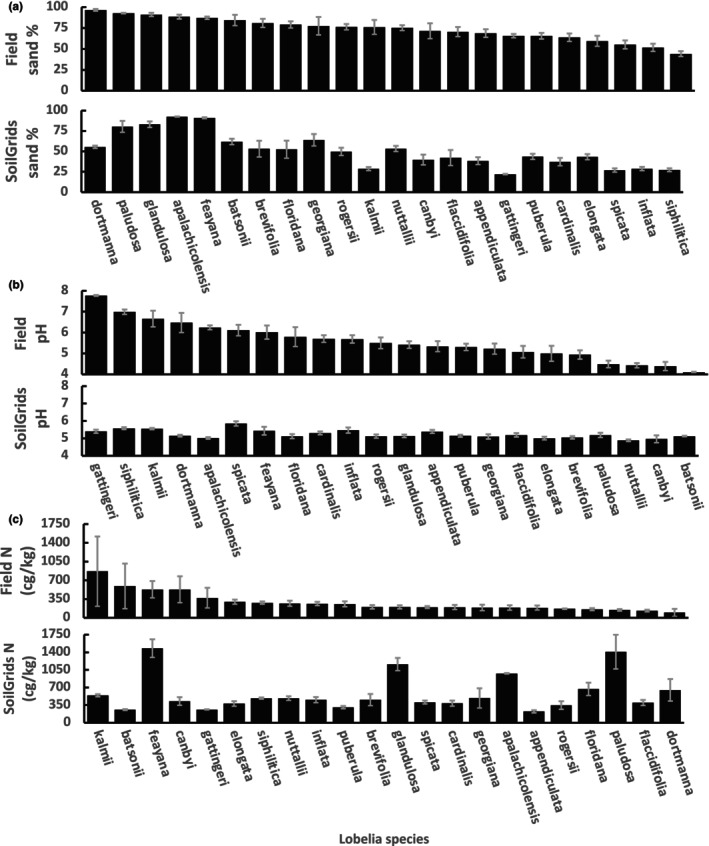
Comparing ranked species means derived from field‐collected soil measurements and SoilGrids predictions. The top graph within each panel shows the mean (± standard error) of measurements on field‐collected soil ranked in order from highest to lowest on the *x*‐axis. The bottom graph within each panel shows the mean (± standard error) of SoilGrids predictions for the variables, while maintaining the same order on the *x*‐axis to compare ranks. (a) % Sand (*r*_S_ = .84), (b) pH (*r*_S_ = .61), (c) % Nitrogen (*r*_S_ = −.06).

Comparing the rank order of the *Lobelia* species, the SoilGrids predictions do not mirror ranks based on field‐collected data. Comparisons for sand, pH, and nitrogen (Figure 2) illustrate strong, medium, and weak correlations between predictions and field data. Spearman correlation tests indicate that the rankings of species means are significantly related for soil texture (clay *r*_S_ = .74, silt *r*_S_ = .79, sand *r*_S_ = .84; all *p* < .0001) and pH (*r*_S_ = .61, *p* = .002). However, while rankings of species means may be partially consistent, SoilGrids species means do not often reflect true field values. For example, species that affiliate with alkaline soil pH show highly underestimated soil pH means from modeled SoilGrids data (e.g., *L. siphilitica* soils have a mean pH of 7.0 but the SoilGrids estimate is 5.6). In contrast to soil texture and pH, species means for soil C and N calculated from SoilGrids data appear to be completely unrelated to values measured from the field (carbon *r*_S_ = .21, *p* = .35; nitrogen *r*_S_ = −.06, *p* = .79).

The relationships between SoilGrids data and *L. siphilitica* sex ratios did not match relationships between field data and sex ratios (Table 1). Using field data from 30 population sites, percentage of females in a population was positively associated with clay content and negatively associated with sand content. Silt and pH showed no relationship with the percent of females in a population. Using SoilGrids predictions for these same 30 populations, no associations were significant, but clay content and pH were marginally positively associated with female percentage (*p* < .1). When expanding the sample to 195 populations with known sex ratios, the association of modeled SoilGrids clay and sand content became significant, better matching the results from the empirical dataset based on direct measures of both soil and female frequency at 30 population sites.

**TABLE 1:** Associations of population sex ratios of *Lobelia siphilitica* with soil data collected from the field versus predicted from SoilGrids.

Soil variable	A. Field soil samples from population sites (*n* = 30)		B. SoilGrids matching field samples (*n* = 30)		C. SoilGrids matching sites with sex‐ratio data only (*n* = 195)	
	*r*_S_	*p*‐Value	*r*_S_	*p*‐Value	*r*_S_	*p*‐Value
Clay	**.45**	**.01**	.31	.09	**.19**	**<.001**
Silt	.23	.2	−.002	.98	**.37**	**<.0001**
Sand	**−.37**	**.03**	−.23	.2	**−.40**	**<.0001**
pH	.07	.6	.28	.1	.08	.2

*Note*: Spearman's correlation (*r*_S_) and *p*‐values are provided for assessing the relationship between the proportion of females within populations and field‐collected soil samples (A) or SoilGrids predictions (B, C). Significant relationships are shown in bold. (A) Field data from 30 populations where soil samples and sex ratios were both collected. (B) Data from SoilGrids predictions for the same 30 populations as in A. (C) Data from SoilGrids predictions for 195 populations where sex ratios were observed but soil samples were not collected.

## 4 DISCUSSION

Plant distributions are commonly constrained by soil properties (e.g., nutrient availability and water holding capacity), making digital soil maps a potentially valuable resource for improving plant species distribution mapping, forecasting, and making inferences about plant species' niches (Mod et al., 2016; Roe et al., 2022; Velazco et al., 2017; Zuquim et al., 2020). In this study, we explored the utility of SoilGrids for investigating biogeographic patterns within and among species using soil samples from 212 *Lobelia* population sites representing a broad range of habitats. Most datasets that have been used to evaluate SoilGrids predictions are derived from random or systematic soil sampling distributed across a geographic area of interest (Bodenstein et al., 2022; Caubet et al., 2019; Dandabathula et al., 2022; Dharumarajan et al., 2021; Huang et al., 2022; Liang et al., 2019; Radočaj et al., 2023; Tifafi et al., 2018). Our test incorporated constraints that are inherent in “presence” datasets for modeling the distributions of individual species (Jeliazkov et al., 2022). Our study organisms determined the locations of soil sampling sites, introducing constraints on the specific types of habitats sampled and their distribution across the landscape.

Of the six soil variables predicted by SoilGrids, soil texture variables (percent sand, silt, and clay) were most similar to measurements taken on field samples. pH values showed a poor but significant relationship, and soil carbon and nitrogen predictions did not correspond with direct measurements at all. Although the slopes of these relationships were significantly different from 1.0, our analysis indicates that certain SoilGrids variables may be of some usefulness for biogeographic analyses. For example, when comparing edaphic conditions among species, texture and pH may provide a broad indication of species rank‐orders, albeit not actual field values. In our analysis of *L. siphilitica* population sex ratios, we also found that noise in predicted soil texture variables may be overcome by increasing sample size, potentially revealing similar associations as those found using a smaller dataset (Table 1). Although not directly tested in this study, the lack of fit between predicted and actual values is likely to be even greater when population presence information is taken from online databases (e.g., GBIF) rather than taken in situ, as error rates in location data tend to be extremely high across taxa (Zizka et al., 2020). Overall, our results indicate that caution should be exercised, but that using predicted data from SoilGrids may still be helpful in generating hypotheses about the importance of soil texture and pH in species biogeography, as long as the number of accurate presence points is sufficient.

### 4.1 Use of SoilGrids in ecological inference and statistical modeling

Our results have important consequences for using SoilGrids to assess variable importance in constructing species distribution models, mapping habitat suitability, and revealing ecological relationships. Even in cases where modeled predictor variables have a decent relationship with underlying true values (e.g., best shown here for soil texture variables), error in predictor variables leads to lack of statistical power and biased parameter estimates and projections. In some cases, it may be possible to reduce the effects of predictor‐variable uncertainty by taking advantage of spatial autocorrelation and joint species distributions, or by statistically propagating known variance in predictor values as part of the SDM (McInerny & Purves, 2011; Stoklosa et al., 2015). The latter methods may prove useful and should be explored further for SoilGrids data because the database provides a measure of model prediction uncertainty (Poggio et al., 2021).

Problems using SoilGrids variables are likely to remain particularly acute for several common situations. Mismatches between grain size resolution of predictor variable estimates and the scales at which individual organisms or populations respond to the environment are known to be problematic (Moudrý et al., 2023; Moulatlet et al., 2017). As shown here, even the 250 m SoilGrids predictions may not be fine enough resolution to use with species that have small population sizes or species that specialize on soil types that either occur on a small scale or are difficult to predict using a digital soil model.

In addition, if true conditions are poorly reflected by interpolated predictor variables, SDMs can provide misleading inferences, even in cases where algorithms generate a model with high predictive accuracy (Smith & Santos, 2020). We found that SoilGrids frequently failed to predict values that are extreme but not uncommon in soils, or predicted extreme values in incorrect locations. For instance, the extremely low variation in pH estimates from SoilGrids is likely to result in reduced discrimination among sites and lower weighting in an SDM, whereas the increased variation in soil N will likely result in misleading predictions and variable importance. The exceptionally narrow range of soil pH values predicted by SoilGrids at our sampling sites compared to measured values (as well as in Cramer et al., 2019) is particularly problematic given its importance as a driver of variation in nutrient and biotic soil properties.

### 4.2 Comparison to other SoilGrids validation studies

Despite our biogeographically focused sampling design, our results are broadly similar to previous studies that used systematic or random sampling to assess the accuracy of SoilGrids over larger landscape scales. SoilGrids predictions of texture data appear more reliable than predictions of soil carbon and nitrogen, and silt and sand have stronger relationships than clay, including in the cross‐validation performed on the newest iteration of SoilGrids (Poggio et al., 2021). Because the United States contains many soil cores that were used as training data for the SoilGrids algorithm, our study assessed the accuracy of SoilGrids under a favorable scenario, compared to regions with limited training data. The relationships for soil texture found here were similar to those reported in France (Caubet et al., 2019), another area with high density of training data. Results in regions with fewer training points are more variable: no relationships were found between SoilGrids texture predictions and field textures in Norway or Croatia (Huang et al., 2022; Radočaj et al., 2023), whereas results in arid regions in India were similar to what we observed here (Dandabathula et al., 2022). This suggests that biases or noise in SoilGrids predictions of soil texture may be related to regional differences in drivers of soil texture variation rather than the density of training data. Indeed, based on our comparison of the clay fraction, there may be certain ecoregions where SoilGrids predictions would be more suitable for use (e.g., the Ozark/Ouachita Appalachian forests and southeastern USA plains).

Although valuable in global analyses and modeling, the SoilGrids estimates of soil carbon stocks are often found to be inaccurate when compared to direct measurements. We found a very poor relationship between direct measurements of soil carbon and nitrogen contents and estimates in SoilGrids. This finding resembles several other studies finding essentially no relationship (*R* ^2^ < .15) between SoilGrids250 carbon values and independent regional datasets generated using non‐biogeographic sampling approaches in China (Liang et al., 2019) and Western Ghats, India (Dharumarajan et al., 2021). Somewhat better results have been obtained in southern Africa (Bodenstein et al., 2022) and European countries (Tifafi et al., 2018), but these analyses still suggest that extreme caution must be used in using point estimates from SoilGrids as an indicator of soil carbon at any particular location. In addition to limited utility in biogeographic modeling, this may also explain the consistent overestimation of regional carbon stocks by SoilGrids (Duarte et al., 2022; Liang et al., 2019; Silatsa et al., 2020).

## 5 CONCLUSIONS

The importance of suitable soil characteristics in determining plant species presence motivates the use of digital soil predictions for species distribution modeling. Our sampling scheme represents a best‐case scenario for assessing the accuracy of SoilGrids in modeling the environmental conditions associated with widespread, low‐abundance plant species, but we recommend that extreme caution must be used even under these circumstances. Our findings confirm that soil texture variables are often better predicted than chemistry variables, with two additional insights. First, our analysis of *L. siphilitica* sex ratios indicated that having a sufficient number of precise sampling locations appears to be more important for enhancing signal‐to‐noise than having a higher density of training points within a region. Second, while SoilGrids estimates may not reflect actual field values, rank ordering of mean species values may be somewhat reliable from predicted data. Soil texture may be easier to predict because it varies more gradually over time and space compared to chemical properties, which can be extremely dynamic, especially with changes in land use (Guo & Gifford, 2002). Incorporating additional drivers of soil properties (e.g., disturbance, edge effects) into digital soil models may be helpful in improving accuracy of chemical predictions and increase reliability of modeled soil data for uncovering biogeographic patterns.

## AUTHOR CONTRIBUTIONS

**Tony Miller:** Conceptualization (equal); data curation (lead); formal analysis (lead); investigation (lead); methodology (equal); software (lead); validation (equal); visualization (equal); writing – original draft (lead); writing – review and editing (equal). **Christopher B. Blackwood:** Conceptualization (equal); formal analysis (supporting); funding acquisition (equal); investigation (supporting); methodology (equal); resources (supporting); software (supporting); validation (supporting); visualization (equal); writing – original draft (supporting); writing – review and editing (equal). **Andrea L. Case:** Conceptualization (equal); data curation (supporting); formal analysis (supporting); funding acquisition (equal); investigation (supporting); methodology (supporting); project administration (lead); supervision (lead); validation (equal); visualization (equal); writing – original draft (supporting); writing – review and editing (equal).

## CONFLICT OF INTEREST STATEMENT

The authors of this manuscript declare that there are no competing interests.

## Supporting information


Figure S1.
Figure S2.
Table S1.
Table S2.

## Data Availability

The raw data and figures are available to download through Open Science Framework: https://osf.io/wf3ad/?view_only=a91dfa7c9d874776abb0df3396285435.
