# Supplementary material for: Assessing the utility of SoilGrids250 for biogeographic inference of plant populations
Source: Ecol Evol. 2024 Mar 11;14(3):e10986. doi: 10.1002/ece3.10986 (PMC10928252; doi:10.1002/ece3.10986)
Supplement: Supplementary file 1 — Figure S1. Figure S2. Table S1. Table S2. [file ECE3-14-e10986-s001.pdf]

Supplemental Information for:

ASSESSING THE UTILITY OF SOILGRIDS250 FOR BIOGEOGRAPHIC INFERENCE OF PLANT POPULATIONS

Tony Miller, Christopher B. Blackwood, Andrea L. Case

Table of Contents:

**Supp Figure S1**	Page 2
**Supp Figure S2**	Page 3
**Supp Table S1**	Page 4
**Supp Table S2**	Page 10

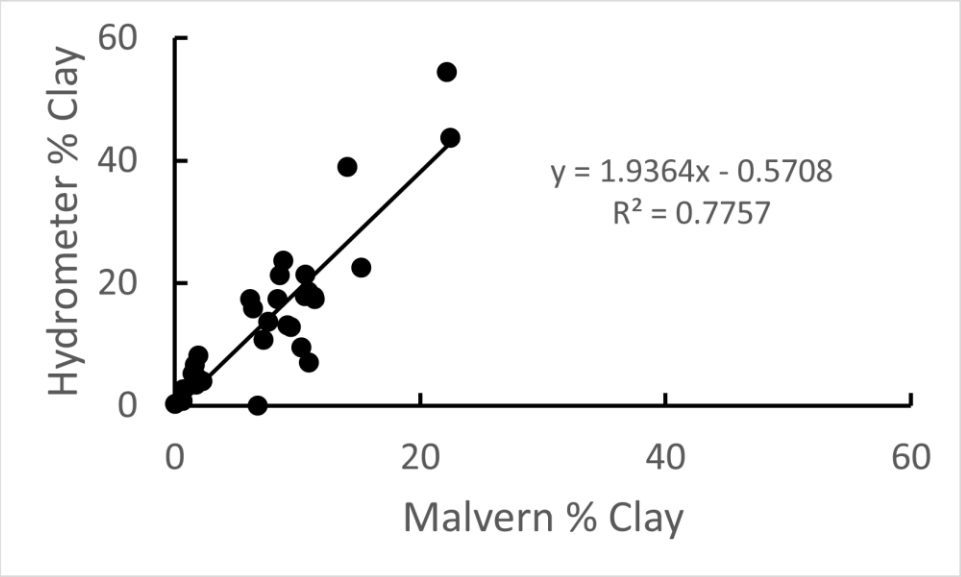

Supp Figure S1: Relationship between clay values obtained from the laser diffraction method (Malvern) and from the sieve hydrometer method (hydrometer)
Clay values for 30 populations were measured using both the laser diffraction method and the sedimentation hydrometer method. Similar to the results found in Di Stefano et al., 2010 (slope=1.9), our data shows a slope of 1.94. We used a multiplier of 1.9 * the laser diffraction method clay value and subtracted the resulting difference from the silt fraction (Di Stefano et al., 2010).

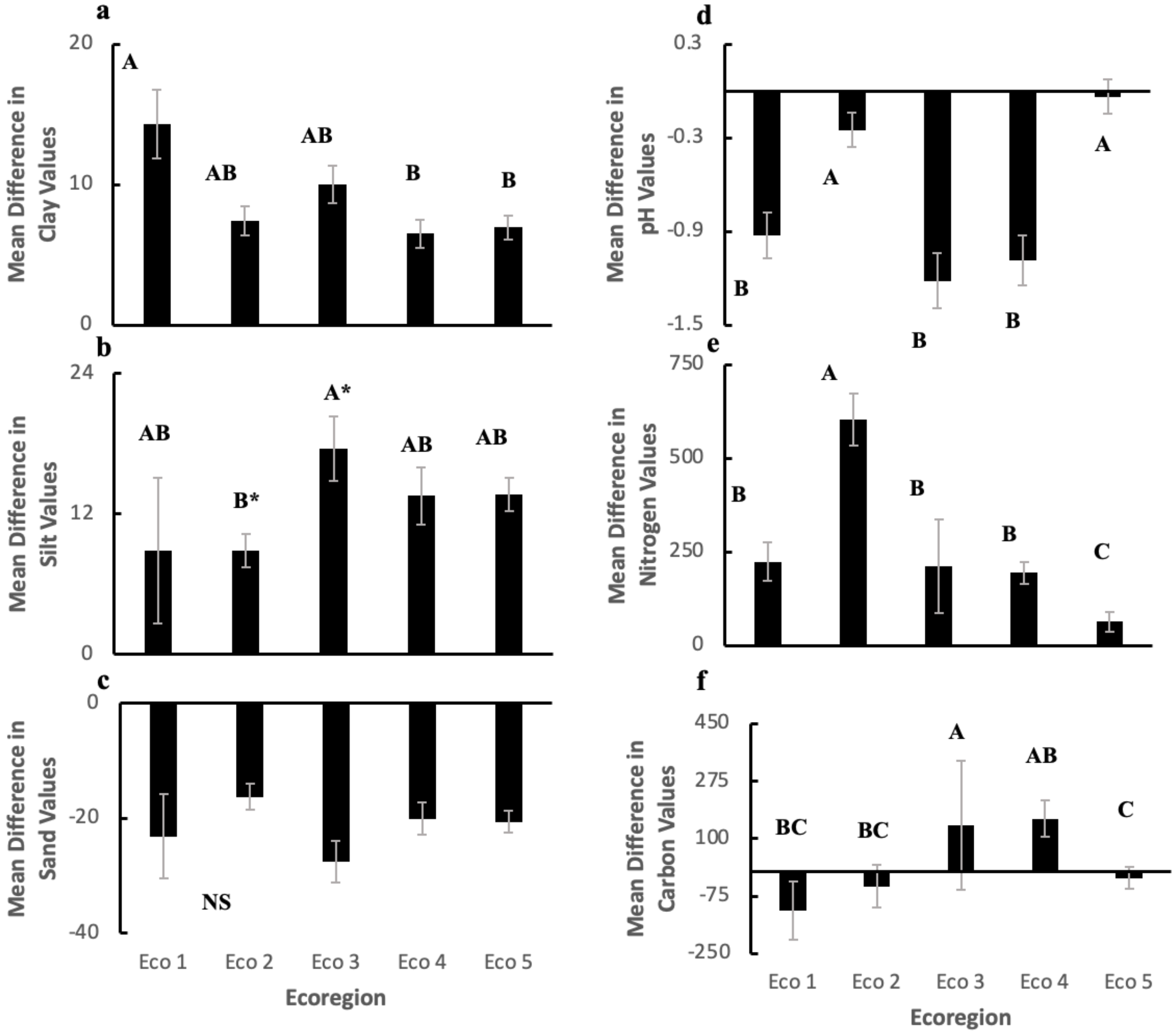

Supp Figure S2: Comparing SoilGrids-measured differences across ecoregions
Difference in mean values (± standard errors) from the 1:1 line representing perfect agreement between field data and SoilGrids predictions, plotted against level-2 ecoregions (key in Table S2). Statistics were derived from nonparametric Kruskal-Wallis one-way ANOVAs and *post hoc* Steel-Dwass tests. (a) Clay is over-predicted across ecoregions, but in ecoregion 1, the clay over-prediction is significantly greater than in ecoregions 4 and 5 ($p<0.01$). (b) The silt fraction is over-predicted across ecoregions, with only marginally significant differences between ecoregions 3 (Steel-Dwass $P=0.0505$). (c) The sand fraction is consistently under-predicted across ecoregions ($p>0.1$). (d) pH is under-predicted in 4 of the 5 ecoregions, most dramatically in ecoregions 1, 3, and 4 ($p<0.0001$). (e) Nitrogen is over-predicted, particularly in ecoregion 2 ($p<0.0001$). (f) Differences in carbon predictions vary in direction and magnitude across ecoregions.

Supp Table S1: Population locations and species information

Site Code	Species	Latitude	Longitude
LAPALBS17132	apalachicolensis	30.08319	-84.5854
LAPALBS17133	apalachicolensis	30.06881	-84.506
LAPALMJ21170CIRCLEL	apalachicolensis	30.18816	-84.2149
LAPALMJ21170CIRCLES	apalachicolensis	30.18816	-84.2149
LAPALMJ21170LARGE	apalachicolensis	30.18816	-84.2149
LAPALMJ21170SMALL	apalachicolensis	30.18816	-84.2149
LAPPEAC21008	appendiculata	31.68738	-92.4068
LAPPEAC21012	appendiculata	32.41368	-94.7113
LAPPEAC21013	appendiculata	34.01998	-95.241
LAPPEAC21016	appendiculata	30.78098	-93.6996
LAPPEAC21020	appendiculata	30.76228	-90.1769
LAPPECB21014	appendiculata	31.16815	-95.9724
LAPPECB21015_MID	appendiculata	29.80322	-94.3868
LAPPECB21018	appendiculata	30.54288	-93.5555
LAPPELD21009	appendiculata	32.65796	-92.0036
LBATSKS17056	batsonii	35.02096	-79.4682
LBATSPA21164	batsonii	35.05629	-79.6191
LBREVAC17112	brevifolia	31.5631	-86.7417
LBREVAC21171	brevifolia	30.27858	-84.8441
LBREVAC21180	brevifolia	30.51783	-89.9558
LBREVBS17075	brevifolia	30.73196	-85.6117
LBREVLD17113	brevifolia	31.7184	-86.6171
LBREVMJ21177	brevifolia	30.40675	-88.6405
LCANBAC21060	canbyi	33.23565	-79.6067
LCANBCB21052	canbyi	38.80125	-75.4234
LCANBCH17102	canbyi	35.60877	-85.897
LCANBGG21059	canbyi	33.21409	-79.7832
LCARDAC21046	cardinalis	38.96173	-77.6134
LCARDAC21047	cardinalis	37.66441	-77.5492
LCARDAC21181	cardinalis	30.81365	-91.3755
LCARDAC21184	cardinalis	30.28602	-94.191
LCARDCB21052	cardinalis	38.80125	-75.4234
LCARDCB21064	cardinalis	34.0462	-81.1895
LCARDCH17104	cardinalis	35.5126	-85.9468
LCARDLD17095	cardinalis	39.1389	-86.3999
LCARDMJ21179	cardinalis	30.56776	-88.6186
LCARDMJ21183	cardinalis	31.00471	-93.2419
LCARDMJ21186	cardinalis	30.09636	-97.2202

LCARDPR17088	cardinalis	44.74035	-68.0135
LCARDRY17145	cardinalis	36.64911	-84.3512
LCARDST17131	cardinalis	42.71266	-71.5482
LDORTAF17077	dortmanna	46.87194	-87.9024
LDORTAF17079	dortmanna	46.8828	-87.9015
LDORTCB21050	dortmanna	41.69838	-71.7716
LELONAC17136	elongata	35.60317	-79.0868
LELONAC17138	elongata	35.36781	-78.3016
LELONAC21058	elongata	32.75521	-80.3318
LELONAC21060	elongata	33.23355	-79.6078
LELONAC21063	elongata	33.23198	-79.482
LELONAC21065_SMALLPLANTS	elongata	34.04969	-81.1788
LELONAC21065LARGEPLANTS	elongata	34.04969	-81.1788
LELONCB21055	elongata	36.58923	-76.0406
LELONCB21064	elongata	34.0462	-81.1895
LFEAYAC21001	feayana	29.60858	-82.2333
LFEAYAC21002	feayana	29.40304	-82.691
LFEAYAC21005	feayana	26.59886	-81.3766
LFEAYLD21004	feayana	27.78587	-82.2448
LFEAYLD21006	feayana	28.18107	-81.4564
LFLACBS17080	flaccidifolia	30.66887	-84.3057
LFLACBS17081	flaccidifolia	30.72407	-86.7916
LFLACCB21015_ESIDE	flaccidifolia	29.80322	-94.3868
LFLACCB21015_MIDDLE	flaccidifolia	29.80322	-94.3868
LFLACCB21015_WSIDE	flaccidifolia	29.80322	-94.3868
LFLACCB21018	flaccidifolia	30.54288	-93.5555
LFLACLD17116	flaccidifolia	31.61028	-86.6353
LFLORBS17124	floridana	30.29526	-85.2565
LFLORBS17125	floridana	30.29537	-85.133
LFLORCB21019_SITE1	floridana	30.39025	-89.9358
LFLORCB21019_SITE2	floridana	30.39025	-89.9358
LFLORCB21021	floridana	30.48721	-88.4572
LFLORCB21022	floridana	31.02387	-86.0193
LGATTCH17074	gattingeri	36.10184	-86.5311
LGATTCH17078	gattingeri	36.07987	-86.5892
LGEORAC17109	georgiana	31.89033	-85.7568
LGEORLD17108	georgiana	33.5425	-85.6485
LGEORMJ21172	georgiana	30.3896	-84.7893
LGEORMJ21173	georgiana	30.31639	-84.7708

LGLAN?MJ21170	glandulosa	30.17105	-84.2065
LGLANAC21026	glandulosa	28.48875	-81.1294
LGLANAC21060	glandulosa	33.2351	-79.6074
LGLANAC21165	glandulosa	31.04912	-81.5527
LGLANAC21166	glandulosa	28.88228	-81.1322
LGLANAC21167	glandulosa	27.70786	-80.9254
LGLANAC21169	glandulosa	29.07171	-82.6449
LGLANAC21171	glandulosa	30.27858	-84.8441
LGLANBS17147	glandulosa	30.41961	-83.6582
LGLANBS17148	glandulosa	30.05252	-85.0709
LGLANBS17149	glandulosa	30.06336	-85.0598
LGLANMJ21173	glandulosa	30.31639	-84.7708
LGLANPA21026	glandulosa	28.43317	-81.1066
LGLANPA21029	glandulosa	30.12767	-81.546
LGLANPA21168	glandulosa	26.54474	-81.8134
LINFLAC21033	inflata	36.08515	-84.7544
LINFLAC21035	inflata	37.3757	-80.524
LINFLAC21039	inflata	42.95769	-73.7714
LINFLAC21046	inflata	38.96173	-77.6134
LINFLBS17089	inflata	44.23826	-88.2946
LINFLCH17102	inflata	35.6081	-85.8972
LINFLRY17145	inflata	36.64911	-84.3512
LINFLSH21036	inflata	39.49313	-78.6683
LINFLSH21038	inflata	42.08645	-76.2803
LINFLSH21042	inflata	41.042	-74.7417
LKALMAC17090	kalmii	41.21231	-81.3682
LKALMAC21043_2KMFROMSIPH	kalmii	41.00241	-74.9177
LKALMAC21043_ISO	kalmii	41.00241	-74.9177
LKALMSH21040	kalmii	43.29513	-73.595
LNUTTAC17107	nuttallii	34.0828	-85.8315
LNUTTAC21028_SITE1	nuttallii	31.947	-81.4529
LNUTTAC21028_SITE2	nuttallii	31.9425	-81.4538
LNUTTAC21044	nuttallii	38.9883	-74.9134
LNUTTAC21048	nuttallii	34.12866	-78.0395
LNUTTAC21049	nuttallii	33.4298	-79.2934
LNUTTAC21054	nuttallii	36.93613	-76.555
LNUTTBS17075	nuttallii	30.73209	-85.6113
LNUTTBS17076	nuttallii	30.55862	-85.8687
LNUTTBS17082	nuttallii	30.76463	-86.9957

LNUTTCB21055	nuttallii	35.02477	-79.4901
LNUTTCB21057	nuttallii	34.06538	-80.1508
LNUTTGG21059	nuttallii	33.21409	-79.7832
LNUTTGG21062	nuttallii	33.24578	-79.5352
LNUTTPA21164	nuttallii	35.05629	-79.6191
LNUTTSH21045	nuttallii	38.75265	-75.7173
LPALUAC21007	paludosa	30.78403	-83.0014
LPALUAC21026_SITE1	paludosa	28.47914	-81.1213
LPALUAC21026_SITE2	paludosa	28.47731	-81.127
LPALUAC21027	paludosa	30.9906	-81.8254
LPUBEAC17138	puberula	35.36858	-78.3021
LPUBEAC17141	puberula	36.57597	-78.5478
LPUBEAC17146	puberula	36.70129	-84.1535
LPUBEAC21051	puberula	39.06203	-75.5552
LPUBEAC21054	puberula	36.93613	-76.555
LPUBEAC21163	puberula	35.45987	-79.9598
LPUBEAC21182	puberula	30.94265	-91.2041
LPUBECB21052	puberula	38.80125	-75.4234
LPUBECB21053	puberula	37.11474	-77.2393
LPUBECB21061	puberula	33.2378	-79.5384
LPUBECH17102	puberula	35.6081	-85.8972
LPUBEGG21062	puberula	33.24578	-79.5352
LPUBEJR17139	puberula	35.88822	-79.0164
LPUBELD17101	puberula	35.4512	-86.0304
LPUBELD17105	puberula	33.9761	-86.2571
LPUBELD17109	puberula	31.89033	-85.7568
LPUBEMJ21175	puberula	31.07514	-86.5164
LPUBEMJ21183	puberula	31.00471	-93.2419
LPUBEMJ21185	puberula	29.52118	-96.609
LPUBERY17145	puberula	36.64911	-84.3512
LROGEAC17112	rogersii	31.5631	-86.7417
LROGEAC21176	rogersii	30.88062	-86.9542
LROGEAC21178	rogersii	30.57255	-88.6396
LROGEAC21180	rogersii	30.51783	-89.9558
LROGELD17111	rogersii	32.5758	-86.7999
Apple Creek	siphilitica	40.6782	-81.8421
Bend View I	siphilitica	41.45335	-83.7907
Blue Bend	siphilitica	37.92093	-80.266
Bonnivale	siphilitica	39.17028	-81.4897
Brushy Farm	siphilitica	39.21862	-80.9661

Buckwheat	siphilitica	39.53052	-79.6364
Buttermilk Falls	siphilitica	42.40128	-76.5132
Campbell Prairie	siphilitica	41.53586	-83.8395
Chenango Valley	siphilitica	42.2052	-75.8381
Ellenboro Hike Bike	siphilitica	39.26413	-81.0584
Ernst Bike Trail	siphilitica	41.5204	-80.0556
Furstenberg Nature Area	siphilitica	42.28086	-83.7093
Gahanna Woods	siphilitica	39.88797	-82.797
George R. Clark	siphilitica	39.91443	-83.9069
Hardy Dam	siphilitica	43.48456	-85.6388
Jennings Woods	siphilitica	41.17188	-81.2018
Kaylor Road	siphilitica	40.60705	-81.5992
Letchworth	siphilitica	42.65542	-77.9692
LSIPHAC17096	siphilitica	39.22021	-86.3425
LSIPHAC17117	siphilitica	39.51021	-84.7181
LSIPHAC17142	siphilitica	37.27462	-80.312
LSIPHAC17146	siphilitica	36.70129	-84.1535
LSIPHAC21043_2KMFROMKALMII	siphilitica	41.00241	-74.9177
LSIPHAC21043_ISO	siphilitica	41.00241	-74.9177
LSIPHAC21046	siphilitica	38.96173	-77.6134
LSIPHLD17095	siphilitica	39.1384	-86.3991
LSIPHRY17145	siphilitica	36.65066	-84.3492
LSIPHTM17073	siphilitica	41.63923	-83.7913
LSIPHTM17128	siphilitica	41.46071	-80.0124
LSIPHTM17129	siphilitica	41.52278	-80.0734
LSIPHTM17130	siphilitica	41.17196	-81.2016
LSIPHTM17150	siphilitica	41.45846	-80.0195
LSIPHTM17219	siphilitica	41.53458	-83.8395
Luton Park	siphilitica	43.11401	-85.5225
Mountwood Park	siphilitica	39.24511	-81.3081
Munroe Falls Metropark	siphilitica	41.12899	-81.4192
Mystery Hole	siphilitica	38.1274	-81.1422
Olive Green	siphilitica	39.74376	-81.6153
Pearson	siphilitica	41.64054	-83.4347
Quail Hollow	siphilitica	40.97852	-81.3021
Rt. 62/60	siphilitica	40.48667	-81.9784
Smithville	siphilitica	40.8627	-81.8541
Storm Drain	siphilitica	38.14348	-81.1572
Wabash-Cannonball	siphilitica	41.55639	-83.854
Yellowwood 1	siphilitica	39.21913	-86.3425

LSPICAC17073	spicata	41.63718	-83.7922
LSPICAC21033	spicata	36.08515	-84.7544
LSPICAC21041_GLENFALLS	spicata	43.28974	-73.6122
LSPICAC21043	spicata	41.00241	-74.9177
LSPICAC21046	spicata	38.96173	-77.6134
LSPICCB17072	spicata	41.5063	-83.799
LSPICCB21011	spicata	36.8592	-95.1145
LSPICDB17084	spicata	40.1518	-89.843
LSPICDB17085	spicata	40.74345	-88.6132
LSPICDB17086	spicata	40.44516	-88.0977
LSPICDB17087	spicata	40.81082	-89.5905
LSPICSH21034	spicata	35.91186	-85.1232
LSPICSH21036_LARGEFRUITS	spicata	39.49313	-78.6683
LSPICSH21036_WV	spicata	39.49313	-78.6683
LSPICSH21042	spicata	41.03624	-74.7479

Supp Table S2: Ecoregions used in the current study

*Asterisk indicates ecoregions was not used in analysis due to low sample size.

Level 2 Ecoregion	Ecoregion Coding	# Sites Sampled
Central USA Plains	Eco 1	16
Mississippi Alluvial and Southeast USA Coastal Plains	Eco 2	63
Mixed Wood Plains	Eco 3	22
Ozark/Ouachita Appalachian Forests	Eco 4	35
Southeastern USA Plains	Eco 5	68
Mixed Wood Shield	Eco 6	3*
Temperate Prairies	Eco 7	1*
Texas-Louisiana Coastal Plain	Eco 8	4*
